# Efficacy and safety of SGLT2 inhibitors in patients with heart failure: A protocol for systematic review and meta-analysis

**DOI:** 10.1097/MD.0000000000028636

**Published:** 2022-01-21

**Authors:** Li Wang, Xiaoning Guo

**Affiliations:** a Department of Emergency, Beijing Friendship Hospital, Capital Medical University, Beijing, China; b Department of Internal Medicine, Beijing Yide Hospital, Beijing, China.

**Keywords:** efficacy, heart failure, meta-analysis, sodium-glucose transporter 2

## Abstract

**Background:**

Heart failure (HF) prognosis without therapy is poor, however introduction of a range of drugs has improved it. We aimed to perform a protocol for systematic review and meta-analysis on the effects and safety of Sodium-Glucose Transporter 2 inhibitors in HF patients.

**Methods:**

This protocol of systematic review and meta-analysis has been drafted under the guidance of the preferred reporting items for systematic reviews and meta-analyses protocols. This study will use the PubMed, Cochrane Library, Embase, Web of Science, and Medline databases. In addition, we will also collect 4 databases of China: China National Knowledge Infrastructure, China Biomedical Literature Database, China Science Journal Database, and Wan-fang Database. The risk of bias of included studies is estimated by taking into consideration the characteristics including random sequence generation, allocation concealment, blinding of patients, blinding of outcome assessment, completeness of outcome data, selective reporting and other bias by Cochrane Collaboration's tool. All analyses were performed with Review Manager (RevMan) software, version 5.3 (Update Software Ltd, Oxford, Oxon, UK).

**Results:**

The results of this systematic review and meta-analysis will be published in a peer-reviewed journal.

**Conclusion:**

Sodium-glucose transporter 2 inhibitors may improve critical outcomes in HF patients, and it is apparently safe.

**Open Science Framework registration number:**

https://doi.org/10.17605/OSF.IO/ZKE3Y 10.17605/OSF.IO/MP5SD

## 1 Introduction

Heart failure (HF) is a syndrome characterised by symptoms (such as breathlessness, ankle swelling, and fatigue) and signs (eg, raised jugular venous pressure, pulmonary crackles, and peripheral edema) caused by structural or functional cardiac abnormalities that lead to elevated intracardiac pressures or a reduced cardiac output at rest or during stress.^[1–3]^ HF is a leading and increasing cause of morbidity and mortality worldwide. The prevalence of HF is age-dependent, ranging from less than 2% of people younger than 60 years to more than 10% of those older than 75 years.^[1,4]^ As a result of ageing of the general population and improved treatment of acute cardiovascular events, the prevalence of heart failure is projected to increase by 25% in the next 20 years.^[5]^ The prognosis of HF without therapy is poor, however the introduction of a range of pharmacological treatments has improved it. New drugs have been studied looking for better outcomes, such as neprilysin inhibitors and ivabradin, and some of them have been incorporated in guidelines.

Sodium-Glucose Transporter 2 (SGLT2) inhibitors increase the urinary excretion of glucose, allowing a reduction of glycaemia, and are recommended in patients with type 2 diabetes.^[6,7]^ Currently, some studies have described potential protective effects against progressive renal events and hospitalization rates due to HF.^[8]^ Some authors have proposed that SGLT2 inhibitors promotes the fasting transcriptional paradigm, and increases ketone bodies, which changes the myocardial metabolism and raises antioxidant and anti-inflammatory effects.^[9]^

Randomized controlled trials (RCTs) have evaluated the effects of SGLT2 inhibitors in patients with HF for improving symptoms, mortality, hospitalization, and biomarkers, however results are heterogeneous. It is relevant to synthesize the current evidence in order to improve the evidence-based decision making in clinical practice. Therefore, we performed a protocol for systematic review and meta-analysis to assess the efficacy and safety of SGLT2 inhibitor in patients with HF.

## 2 Methods

### 2.1 Protocol register

This protocol of systematic review and meta-analysis has been drafted under the guidance of the preferred reporting items for systematic reviews and meta-analyses protocols.^[10]^ It has been registered on open science framework (Registration number: 10.17605/OSF.IO/MP5SD). Ethical approval is not required for this study since it relies on secondary data.

### 2.2 Search strategy

This study will use the PubMed, Cochrane Library, Embase, Web of Science, and Medline databases. In addition, we will also collect 4 databases of China: China National Knowledge Infrastructure, China Biomedical Literature Database, China Science Journal Database, and Wan-fang Database. We will consider articles published between database initiation and December 2021. Two authors will independently draft and carry out the search strategy. In addition, we manually retrieve other resources, including the reference lists of identified publications, conference articles, and gray literature. The key terms used for the search were (“SGLT2” or “Sodium-glucose cotransporter-2 inhibitors” or “canagliflozin” or “dapagliflozin” or “empagliflozin” or “ertugliflozin”) and “heart failure.”

### 2.3 Inclusion and exclusion criteria

(1) Participants: Patients were diagnosed of HF; (2) Intervention: The experimental group was treated with SGLT2 inhibitors, and the control group was treated with the standard regime, with no restrictions on the dose and course of treatment; (3) Outcome measures: (I) the risk of all-cause mortality; (II) left ventricular ejection fraction; (III) hospitalization for HF; (IV) occurrence of adverse reactions; (4) Study type: RCTs. The exclusion criteria for this study were as follows: (I) duplicate studies, experience summaries, case reports, reviews; (II) studies with incomplete information or animal experiments; (III) studies with fewer than 15 cases; (IV) the loss rate of the subjects was >20%.

### 2.4 Study selection

Two authors independently reviewed all titles and abstracts of studies identified by the above searches. Full texts of any potentially useful studies were reviewed, and disagreements were resolved by discussion

### 2.5 Data extraction

The following data were extracted for each article: (1) bibliographical data, including authors and year of publication; (2) clinical trial features such as sample size, study flow, recruitment method, criteria for inclusion and exclusion, primary measures, time and point of assessments, and duration of the intervention; (3) participant characteristics such as age, sex, and so on; (4) patient background, including country and race; and (4) study drop-out rate and handling of missing data.

### 2.6 Risk of bias assessment

Two investigators will separately assess the risk of bias of the included studies using the Cochrane risk of biasassessment tool. The evaluation of each study mainly included the following seven aspects: random sequence generation, allocation hiding, blinding of participants and personnel, blinding of outcome assessment, incomplete outcome data, incomplete outcome data, selective outcome reporting, and other biases. Finally, the bias of the study will be rated on three levels: “low”, “high”, and “ambiguous”. These even domains will be separately appraised by two reviews, and discrepancies will be addressed by consulting a third reviewer.

### 2.7 Statistical analysis

Statistical heterogeneity was explored using a chi-square test and expressed as an *I*^2^ index (a significance level of *P* < .10). *I*^2^ values over 50% represented substantial heterogeneity. If there was no heterogeneity, a fixed-effects model was used for meta-analysis; otherwise, a random-effects model was used. Continuous variables were expressed as the weighted mean difference or standardized mean difference and 95% confidence interval. Weighted mean difference was used when data were measured in the same scale and standardized mean difference were used if data were measured using different scales. Potential publication bias was tested with a funnel plot. To evaluate publication bias, we will construct a funnel plot if the number of included studies is sufficient. All analyses were performed with Review Manager (RevMan) software, version 5.3 (Update Software Ltd, Oxford, Oxon, UK).

## 3 Discussion

Prior RCTs have evidenced that SGLT2 inhibitors have beneficial effects on hospitalization and cardiovascular events, including mortality. SGLT2 inhibitors mechanism of action in HF patients has not been completely elucidated. Despite its well-known effect as a glycosuric agent, there are other proposed explanations for benefits. Some of them are metabolic changes, such as more ketone body production, activation of anti-inflammatory and anti-oxidative pathways, and reduction of advanced glycation end-products mediated effects.^[11–13]^ SGLT2 inhibitors reduces Nod-like receptor protein 3 (NLRP3) inflammasome activation, reducing IL1-β in macrophages, which is associated with less development of atherosclerotic plaques.^[14]^ However, the efficacy and safety of SGLT2 inhibitors for the treatment of HF remains under debate. To the best of our knowledge, this is the first meta-analysis of the effect of SGLT-2 inhibitors on HF patients. The results of our meta-analysis will evaluate whether SGLT-2 inhibitors are beneficial for HF patients, providing evidence regarding the clinical use of SGLT-2 inhibitors in these patients.

## Author contributions

**Conceptualization:** Li Wang.

**Funding acquisition:** Xiaoning Guo.

**Investigation:** Li Wang.

**Methodology:** Li Wang.

**Writing – original draft:** Li Wang.

**Writing – review & editing:** Xiaoning Guo.
